# Economic Burden of Road Traffic Injuries in Nepal

**DOI:** 10.3390/ijerph17124571

**Published:** 2020-06-25

**Authors:** Amrit Banstola, Jesse Kigozi, Pelham Barton, Julie Mytton

**Affiliations:** 1 Faculty of Health and Applied Sciences, University of the West of England, Bristol BS16 1QY, UK; julie.mytton@uwe.ac.uk; 2 Health Economics Unit, University of Birmingham, Birmingham B15 2TT, UK; J.Kigozi@bham.ac.uk (J.K.); p.m.barton@bham.ac.uk (P.B.)

**Keywords:** economic burden, economic impact, road traffic injuries, road traffic crashes, Nepal

## Abstract

The evidence of the economic burden of road traffic injuries (RTIs) in Nepal is limited. The most recent study, conducted in 2008, is now considered outdated because there has been a rapid increase in vehicle numbers and extensive road building over the last decade. This study estimated the current economic costs of RTIs in Nepal, including the direct costs, productivity costs, and valuation of pain, grief, and suffering. An incidence-based cost-of-illness analysis was conducted from a societal perspective, employing a bottom-up approach using secondary data. All costs incurred by the patients, their family members, and costs to society were estimated, with sensitivity analyses to consider uncertainty around the data estimates available. Productivity loss was valued using the human capital approach. The total costs of RTIs in 2017 were estimated at USD 122.88 million. Of these, the costs of productivity loss were USD 91.57 million (74.52%) and the pain, grief, and suffering costs were USD 18.31 million (14.90%). The direct non-medical costs were USD 11.50 million (9.36%) whereas the direct medical costs were USD 1.50 million (1.22%). The economic costs of RTIs increased by threefold since 2007 and are equivalent to 1.52% of the gross national product, indicating the growing national financial burden associated with preventable RTIs.

## 1. Introduction

Road traffic injuries (RTIs) are a key injury burden worldwide. According to the Global Burden of Diseases (GBD) data for Nepal, RTIs are the leading cause of death among people aged 15 to 49 years [1]. RTIs led to 296,686 years of potential life loss because of premature deaths and accounted for 36% of the total injury-related disability-adjusted life years (DALYs) lost in 2017 [1]. RTIs were also the fifth leading cause of DALYs for all age groups in the same period.

RTIs cause not only premature deaths and disabilities but also considerable costs to victims and their families [2,3]. There is evidence that the costs associated with RTIs can lead to impoverishment [4,5]. RTIs affect the national economy as the cost of RTIs have been estimated by the World Health Organisation (WHO) to account for 1.0% to 2.0% of the gross national product (GNP) for each country, each year [6]. The cost mainly arises from premature death; the costs of treatment, long-term care, and rehabilitation; and the inability of those with severe disabilities to work to their full capacity [7].

The evidence of the economic burden of RTIs in Nepal is limited. A few global-level studies estimated the economic burden of RTIs for Nepal [8,9]. A study by Dalal et al. showed that Nepal lost an estimated 6.30% of its gross domestic product (GDP) due to RTIs in 2005 [8]. Another estimate shows that between 2015 and 2030, RTIs will cost Nepal USD 778 million (0.167% of its total GDP) [9]. There have been only two national-level studies [10,11]. The first study that estimated the cost of RTIs was conducted in 1996 and used data from the fiscal year 1995/96 [10]. The study reported a total cost of RTIs in Nepal of USD 13.58 million in 1996, as stated by ND LEA et al. [11]. This study was updated in 2008 using alternative methods to calculate productivity losses. Using data from the fiscal year 2006/07, the total cost for RTIs was estimated to be USD 40.65 million in 2007 [11].

These national-level studies are now considered outdated because, since 2008, there has been a rapid increase in the use of motor vehicles and extensive road-building programmes that have shifted the ranking of RTIs from the eighth to the seventh leading cause of death in Nepal [1]. The number of registered vehicles has increased from 710,914 in the fiscal year 2007/08 to 3,221,042 in the fiscal year 2017/18 [12], and an estimated 10,921 km of new roads have been built by central and provincial agencies in the five years between fiscal year 2013/14 and fiscal year 2017/18 [13,14].

A small number of local studies have attempted to measure the costs of RTIs [15,16,17]. A study by Joshi and Shrestha on 505 injury patients showed out-of-pocket (OOP) expenses per patient of USD 44.60 in 2008, though only 42.5% of participants were patients who had experienced an RTI [15]. Saito et al. found that in Kathmandu (capital of Nepal), injuries, particularly those resulting from RTIs, were among the top ten causes of catastrophic household expenditure on health in 2014 [16]. A study by Sapkota and colleagues of 100 victims of motorbike crashes and their caregivers’ reported OOP expenses per patient of USD 1224 in 2015 [17]. These studies are relatively small in size, conducted in a limited number of health facilities, in a specific geographical area of a country, focused on motorbike RTIs only, had limited cost items, and tended to poorly report the methods for estimating costs incurred, raising the issue of whether it is appropriate to generalise these findings to the national level. In light of this limited evidence, this study aimed to adopt robust methods and include all available cost items to estimate the economic burden of RTIs in Nepal in 2017.

## 2. Materials and Methods

### 2.1. Study Design

A cost-of-illness (COI) analysis was adopted to estimate the economic burden of RTIs in Nepal. The COI analysis identifies and measures all the costs resulting from a specific injury to society [18,19] and is either based on estimates of the prevalence [20,21,22,23,24] or incidence [25,26,27,28,29,30] of RTIs. In this study, an incidence-based COI approach was used that included the present value of lifetime earnings resulting from all new cases of RTI that occurred in a particular year [19,31]. The study was conducted from a societal perspective, as it sought to encompass the views of all stakeholder groups within society [32]. In this study, a bottom-up costing approach was employed, which measured the amount of each resource that was used and assigned the unit cost of each resource to generate total healthcare costs [32].

### 2.2. Data Sources

#### 2.2.1. Injury Data

The annual incidence of RTIs was obtained from Nepal Police records [33]. According to the Nepal Police, 10,178 road crashes occurred in the fiscal year 2016/17 that resulted in 2384 deaths, 4250 serious injuries, 8290 minor injuries, and 7708 occurrences of vehicle damage. In Nepal, the traffic Police define road death as any person killed immediately or within 30 days of a road crash. Any person injured who is unconscious after the crash is categorised as a person seriously injured, and any person injured excluding persons killed or seriously injured is a minor injury. The average age at the time of injury due to RTI and age at death due to road crashes in 2017, as per the Nepal Police record, was 30 years [33]. The average length of inpatient stay for a person seriously injured in road crashes (17 days) was taken from a previous costing study of RTI in Nepal [11]. In the absence of national data, the duration of absence from work due to RTIs (three days for minor injuries and 20 days for serious injuries) and a number of days spent by a caregiver providing care (eight days for serious injury) was obtained from a study conducted in Bangladesh [34]. The percentage of RTI victims with long-term disabilities in Nepal was obtained from a study by Zafar et al. [35]. According to this study, long-term disabilities occur in approximately 21.1% of non-fatal RTIs in Nepal.

#### 2.2.2. Cost Data

This study included direct medical and direct non-medical costs, productivity losses, as well as pain, grief, and suffering costs, as recommended by the guidelines for estimating the cost of road crashes in low- and middle-income countries (LMICs) [34,36]. The cost items to include in those cost categories were identified from published literature [37,38]. The unit costs per inpatient bed-day (without medication, diagnostic tests, and physician costs) and per outpatient visit were obtained from the World Health Organization CHOosing Interventions that are Cost-Effective (WHO-CHOICE) estimation of hospital costs for Nepal [39]. The average costs of medication and each of the commonly used diagnostic services viz. (i) X-ray, (ii) blood and urine investigations, (iii) Computerized Tomography (CT) scan, and (iv) Electrocardiogram (ECG) were obtained from a hospital-based cross-sectional study on injured patients in Nepal [15]. Although this study was conducted a decade ago, it is the most recent study to date that examined diagnostic costs associated with injuries in Nepal. The unit cost of the doctors’ fee per patient per day was based on expert opinion [40].

The average cost of two-way transportation was obtained from a household survey on a sample of RTI casualties conducted by the Nepal Injury Research Centre (NIRC) in 2018. The average food cost per day (USD 2.39) was based on a consensus of the researchers working in the NIRC. The unit cost of vehicle damage and administrative costs were obtained from a previous costing study of RTI in Nepal [11]. Due to the absence of data on an annual income/daily wage rate of persons injured in road crashes, the national average annual per capita income/daily wage rate was considered similar to other studies [24,30,41]. The national average annual per capita income and daily wage rate were obtained from the Nepal Living Standard Survey 2010/2011 [42].

### 2.3. Cost Analysis

Any unit costs from other years were first converted into 2017 values in Nepalese Rupees (NRs) adjusting for inflation [43] using the GDP deflator of Nepal [44] and were then converted to US dollars (USD) using the exchange rate of NRs. 104.512 = USD 1 for 2017 [45]. All costs in this study are presented in 2017 USD. The number of RTI cases was multiplied by the appropriate unit cost, taking account of the number of days in a hospital, where relevant, to produce costs for each cost item.

### 2.4. Cost Calculation

#### 2.4.1. Direct Medical Costs

Direct medical costs included the costs of inpatient services, outpatient services, diagnostic services, medication costs, and doctors’ fees. It was assumed that serious injuries would require inpatient services while both serious and minor injuries would require outpatient services. This is because a seriously injured patient may need outpatient visits following hospital discharge for follow-up and recovery. Serious RTIs also require diagnostic tests following a hospital admission and this study included X-rays, blood and urine investigations, CT scan, and ECG, similar to other studies on the cost of RTIs [2,23,46]. It was assumed that a person seriously injured would require medication per day for the duration of an inpatient stay, whereas a minor injured patient needs one-off medication as they do not require hospital admission. Table 1 shows the base case values including the cost-calculation equations of direct medical costs.

#### 2.4.2. Direct Non-Medical Costs

Direct non-medical costs related to RTIs included transportation costs, caregivers’ food costs, vehicle damage costs, and administration costs. As the food cost of people seriously injured was included within the inpatient service cost, it was not counted here to avoid double counting. Table 2 shows the base case values including the cost-calculation equations of direct non-medical costs. For administration costs, the number of casualties (fatal and non-fatal) [33] was multiplied by the average administration cost per casualty [11]. Due to the lack of more recent data on administration costs and because this cost is not high compared to other cost items as few road crashes are insured and filed in judicial courts in Nepal [11,34], the administrative costs were explored in a sensitivity analysis.

#### 2.4.3. Productivity Loss Costs

Indirect costs regarding productivity loss were estimated as income lost as a result of short-term absenteeism from work, long-term disability, and premature death due to RTIs. Productivity costs resulting from long-term disability and premature death were estimated using the human capital approach (HCA). The HCA estimates the present discounted value of future earnings of an individual assigning monetary value using the wage rate [32]. The average value of productivity loss was calculated as the sum of each future year’s earnings considering the life expectancy [47] converted to a present value using a 3.0% discount rate per year, consistent with WHO recommendations [48]. The amount of time loss of caregivers was also considered because they could have worked had they not been taking care of the seriously injured person [34] and people with long-term disabilities. Table 3 shows the base case values including the cost-calculation equations of productivity costs.

#### 2.4.4. Costs of Pain, Grief and Suffering

The costs of pain, grief and suffering were calculated by assigning 20.0% of the total cost of productivity loss in line with the previous costing study from Nepal [11]. This approach of adding an amount to reflect the cost of pain, grief and suffering using the HCA method is recommended by the guidelines for estimating the cost of road crashes in LMICs [34,36].

### 2.5. Sensitivity Analysis

One-way sensitivity analyses were conducted to deal with the uncertainty surrounding the data in the estimations [32] for these variables:

- Cost per inpatient bed-day, using the cost of tertiary (super-speciality) hospitals in Nepal i.e., USD 5.34 (after adjusted to 2017 value) [39];
- Doctors’ fee per patient in private hospitals (USD 6.22) and government hospitals (USD 1.44) [40];
- Administrative costs (USD 18.65, after adjusted to 2017 value) [11] because these costs are less significant as few road crashes are insured and filed in judicial courts in Nepal [34];
- The discount rate of 10.0% for premature death and long-term disability in line with the previous costing study conducted in Nepal [11];
- Average amount of working years lost due to premature death considering the age at retirement as used by other studies [11,20,24]. The age of retirement in Nepal is 58 years [49].

## 3. Results

The sum of the direct medical and non-medical, productivity loss and pain, grief and suffering costs resulted in a total economic burden of approximately USD 122.88 million in 2017 (Table 4). The total direct costs that included the costs of medical and non-medical costs were estimated at USD 13.0 million and represented 10.58% of the total costs of RTIs in Nepal. The direct non-medical costs that include vehicle damage were responsible for 9.36% whereas direct medical costs accounted for only 1.22% of the total costs of RTIs. The total indirect costs which comprised productivity loss—including the costs of pain, grief, and suffering—were estimated at USD 109.88 million and represented 89.42% of the total costs of RTIs. Of the total costs of RTIs, the productivity costs accounted for 74.52%, followed by the pain, grief, and suffering costs (14.90%).

The direct medical costs of RTIs in 2017 were approximately USD 1.50 million, as shown in Table 4. The greatest share of this cost was medication (45.42%) followed by inpatient services (19.91%). The lowest share was outpatient services, which accounted for only 1.08% of the total direct medical costs of RTIs. Of the total direct non-medical costs (USD 11.49 million), 98.0% was due to vehicle damage. Of the total estimated costs of productivity loss (USD 91.57 million), long-term disability accounted for 58.12%. The cost of pain, grief, and suffering associated with RTIs was estimated to be USD 18.31 million.

### Sensitivity Analysis

The direct medical cost was most sensitive to the cost of the doctors’ fee (Table 5). Applying the doctors’ fee of private hospitals increased the total direct medical costs by 15.44%, whereas using the doctors’ fee of Government hospitals decreased them by 10.29% compared to the base case result. Correspondingly, the direct non-medical cost was most sensitive to the addition of administration costs, and the cost of productivity loss was most sensitive to the use of a 10% discount rate for long-term disability.

## 4. Discussion

This study estimated the total costs of RTIs in Nepal in 2017 as USD 122.88 million. The findings of this study show that the economic burden of RTIs in Nepal increased by threefold (without inflation) over the past decade from USD 40.65 million in 2007 [11] and is nine times higher (without inflation) than the estimates of USD 13.58 million in 1996 [10,11].

### 4.1. Direct Costs

Total direct costs of RTIs in Nepal were estimated at USD 13.0 million, representing 10.58% of the total costs of RTIs; equivalent to USD 1036.45 per injured person. This finding is lower than the costs incurred by motorbike crash victims and their caregivers found in a study from Kathmandu in 2015 (USD 1224 per injured person) [17]. The average cost of RTIs was USD 2596 per injured person in Thailand in 2004 [24], USD 86 in Bangladesh in 2001 [50], and USD 827.6 in India in 2006 [3]. The medical costs represented the lowest share of the total costs in this study (1.22%) and are consistent with other studies [20,21,51]. In Spain, medical costs accounted for 4.0% of the total costs [20] whereas in Australia it was 2.41% [51]. There are differences between the previous study from Nepal [11] and this study in terms of resource use categories, data sources, and estimation approach of costs. For example, the medical costs were limited to the overall costs of medical treatment (rather than cost according to medical services used) in the previous studies, whereas in this study, medical costs incorporated a range of costs including the costs of inpatient services, outpatient services, diagnostic services, doctors’ fees, and medication costs. Similarly, this study included the costs of food and transportation, as well as the costs of caregivers, which were not included in the previous studies. This study, therefore, provides more comprehensive estimates than previous studies.

### 4.2. Indirect Costs

The total indirect costs of RTIs (productivity loss costs and costs of pain, grief and suffering) were estimated at USD 109.88 million, representing 89.42% of the total costs. The higher indirect costs compared to direct costs were similar to the study conducted in Iran [52] and three European countries [23]. This study found that productivity costs were much greater (74.52%) than other cost categories. This finding differed from the previous study from Nepal, which found that a greater share of the economic burden of RTIs was related to vehicle damage followed by productivity loss [11]. The difference could be because of the way by which the discounted value of future years’ earnings was calculated. This study, finding that the costs of productivity loss were much greater than other cost categories, is consistent with studies conducted in other countries [22,23,24,41]. In Thailand, productivity costs lost due to premature death were accountable for 94% of the total cost in 2004 [24]. In Belize, productivity loss accounted for 98.3% of the total economic cost of RTIs in 2007, which was particularly attributed to premature death [41]. The productivity costs were substantial because road deaths occur mostly in young people, resulting in many economically productive years lost.

The total economic costs of RTIs were approximately USD 122.88 million, which amounts to an estimated USD 4.45 for each of the 27.63 million people living in Nepal, and equivalent to 1.52% of the total Nepal GNP for 2017 (USD 8.06 billion) [53]. This finding is consistent with the WHO estimates that the average annual costs of RTIs in LMICs ranges between 1.0% to 2.0% of their GNP [6]. Recent estimates from other South Asian countries for comparison to these estimates for Nepal are limited, however, in Spain, the cost of RTIs was approximately USD 176 for each person in 2004 and represented 1.35% of the GNP [20].

The Nepal Road Safety Action Plan (which has not been promulgated through parliament) recommends a mandatory 10% of the total budget of road construction to be allocated for road safety interventions [54]. In the fiscal year 2017/18, the total cost of road construction in Nepal was USD 856.36 million [55]. The estimates derived from our study would, therefore, suggest that the total cost of RTIs in Nepal is already 1.43 times more than the recommended 10% of road construction costs (USD 85.63 million). The finding also supports the recent World Bank estimates that Nepal will require an additional investment of USD 879 million until 2030 to achieve the sustainable development goals target of a 50% reduction in national road deaths [56].

The costs reported in this study are likely to be a conservative estimate of the economic burden due to some limitations. The data were obtained from different sources, and there is the possibility of each source having some reporting bias. For instance, the study used the road crashes data from the Nepal Police record that is known to be incomplete due to traffic crashes that are not reported to the police [6,34,57]. Generally, a road crash involving at least one moving vehicle on a road that causes death or injury, or vehicle damage should be reported to the Nepal Police. Although many road crashes involving deaths, serious injuries, and major vehicle damage are reported to the police, a large number of crashes involving minor injuries and minor damage to the vehicles are settled at the crash site upon mutual understanding of involved parties without reporting to the police [6,57].

Nepal police officers at the scene of a crash make a judgement on the severity of an injury (either fatal, serious injury, or minor injury) for recording in the official RTI statistics. There are no formal definitions of serious or minor injury, and therefore the police officers make this decision based on personal opinion. This risks misclassification of injury severity and will lead to incomplete reporting of fatal injuries, since some patients with minor or severe injuries may later die from complications arising from the injury. In Nepal, 2384 deaths were recorded by the Nepal Police in 2017 [33] equivalent to 8.6 road deaths per 100,000 population. This figure is lower than the WHO estimate for Nepal of 15.9 per 100,000 population which is in line with WHO estimates for other South Asian countries, e.g., 22.6 per 100,000 for India, 17.4 per 100,000 for Bhutan, 15.3 per 100,000 for Bangladesh, and 14.9 per 100,000 for Sri Lanka [58]. The difference between estimates of death rates from Nepal police records compared to modelled estimates by WHO could be due to the underreporting of road deaths to the traffic police. If WHO estimates are correct, the economic costs due to RTIs could be much higher than the estimates shown by this study.

This study included multiple costs in the cost estimates, but some additional costs were not included or not available for the analysis. For example, the costs of emergency services, surgical services, prosthetic devices, physiotherapy, rehabilitation, follow-up visits, and the costs associated with readmission following an RTI were excluded in the analysis. In Nepal, there is currently a poorly developed emergency medical service, so national data are not available. A further limitation involved the use of the HCA to value productivity loss which has been criticised for overestimating the value of productivity loss as it assigns more value to premature deaths [32]. Other approaches, such as the friction cost approach [20], could have been taken, but the data required were not available. The value of pain, grief, and suffering would be best captured by the WTP approach [59,60,61,62]. However, the data needed for WTP were not available in Nepal at the time of the study. All of these limitations suggest that the total estimates of economic burden are conservative.

### 4.3. Implications for Future Research

These results will be of value in studies modelling the impact of RTIs in Nepal or when estimating the cost-benefit of interventions to prevent RTIs in Nepal. Future research could generate more valid and nationally representative estimates. Specifically, the collection of primary data on costs experienced by patients suffering RTIs would strengthen the data included in the analyses. In addition, a household survey with the RTI victims and their family members could estimate costs associated with minor RTIs not requiring hospital admission, providing data not available in this study.

### 4.4. Implications for Policy

This study provides an up to date estimate of the economic burden of road crashes in Nepal that could be used to advocate for improved road safety in Nepal and be available for future cost-effectiveness studies of interventions to prevent RTIs in Nepal. The high economic burden of RTIs suggests a need for investment in evidence-based, cost-effective multi-sectoral interventions such as the enforcement of speed limits, enforcement of helmet use by motorcycle riders and pillion passengers, seatbelt usage by drivers [63,64], and a comprehensive “safe system” approach [65]. Given the main contributors of the total economic burden of RTIs were costs of long-term disability; premature deaths; and pain, grief, and suffering, policymakers should prioritise crash-related effective post-crash response systems and lifesaving interventions. If the Government of Nepal can spend less of its GNP on the consequences of RTIs, it may have more resources that could be allocated to other sectors and improve the overall economy of the country.

## 5. Conclusions

In conclusion, the study sought to include direct medical, direct non-medical, and productivity loss costs, as well as a valuation of pain, grief, and suffering due to RTIs in Nepal. This study estimated the total costs of RTIs in Nepal at USD 122.88 million in 2017. The total direct medical costs were estimated at USD 1.50 million and direct non-medical costs at USD 11.50 million. Similarly, the total costs of productivity loss were estimated at USD 91.57 million, and the cost of pain, grief, and suffering associated with RTIs was estimated to be USD 18.31 million. The economic costs of RTIs increased by threefold since 2007 and is equivalent to 1.52% of the 2017 GNP, indicating the growing national financial burden associated with preventable road injuries and deaths.

## Figures and Tables

**Table 1:** Base case values and equations for cost calculation (direct medical costs).

Resource Use		Unit	Value	Sources
Cost Items	Measure			
Inpatient services costs (IPC)	*D_ip_* = Average length of inpatient stay	Days	17	[11]
	*C_ip_* = Unit cost per inpatient bed-day *	USD	4.13	[39]
	*N_s_* = Number of people seriously injured requiring inpatient services	Number	4250	[33]
Outpatient services costs (OPC)	*C_op_* = Unit cost per outpatient visit *	USD	1.29	[39]
	*N_i_* = Number of people with injuries requiring an outpatient visit	Number	12,540	[33]
Diagnostic services costs (DGC)	*C_x_* = Average cost of an X-ray *	USD	5.24	[15]
	*N_x_* = Number of X-rays	Number	4250	[33]
	*C_bu_* = Average cost of blood and urine investigations *	USD	9.90	[15]
	*N_bu_* = Number of blood and urine investigations	Number	4250	[33]
	*C_ct_* = Average cost of CT scan *	USD	38.87	[15]
	*N_ct_* = Number of CT scan	Number	4250	[33]
	*C_ecg_* = Average cost of ECG *	USD	0.87	[15]
	*N_ecg_* = Number of ECG	Number	4250	[33]
Doctors’ fee costs (DC)	*D_ip_* = Average length of inpatient stay	Days	17	[11]
	*C_df_* = Average doctors’ fee per patient	USD	3.35	[40]
	*N_sc_* = Number of seriously injured patients checked	Number	4250	[33]
	*N_mc_* = Number of minor injured patients checked	Number	8290	[33]
Medication costs (MC)	*D_ip_* = Average length of inpatient stay	Days	17	[11]
	*C_ms_* = Average cost of medication for serious injury *	USD	8.44	[15]
	*N_sm_* = Number of people seriously injured requiring medication	Number	4250	[33]
	*C_mm_* = Average cost of medication for minor injury *	USD	8.44	[15]
	*N_mm_* = Number of minor injured patients requiring medication	Number	8290	[33]
**Cost-estimation equations (Direct medical costs)**				
$\begin{array}{ll} Cost_{direct\;medical} & =\sum IPC+OPC+DC+MC\\ & =\sum \left(D_{ip}\times C_{ip}\times N_{s}\right)+\left(C_{op}\times N_{i}\right)\\ & +\left\{\left(C_{x}\times N_{x})+(C_{bu}\times N_{bu})+(C_{ct}\times N_{ct})+(C_{ecg}\times N_{ecg}\right)\right\}+\{(D_{ip}\times C_{df}\times N_{sc})\\ & +(C_{df}\times N_{mc})\}+\left\{\left(D_{ip}\times C_{ms}\times N_{sm})+(C_{mm}\times N_{mm}\right)\right\} \end{array}$				

* after adjusted to 2017 value.

**Table 2:** Base case values and equations for cost calculation (direct non-medical costs).

Resource Use		Unit	Value	Sources
Cost Items	Measure			
Transportation costs (TC)	*C_ts_* = Average cost of two-way transportation of serious injury *	USD	8.62	^a^
	*N_s_* = Number of persons seriously injured	Number	4250	[33]
	*C_td_* = Average cost of two-way transportation of those who died *	USD	8.62	^a^
	*N_d_* = Number of persons died in road crashes	Number	2384	[33]
Food costs of caregiver (FC)	*D_ip_* = Average length of inpatient stay	Days	17	[11]
	*C_fc_* = Average cost of food per day per caregiver *	USD	2.39	^b^
	*N_c_* = Number of caregivers of seriously injured persons	Number	4250 ^c^	
Vehicle damage costs (VDC)	*C_vd_* = Average cost of vehicle damage *	USD	1462.10	[11]
	*N_vd_* = Number of vehicle damage	Number	7708	[33]
**Cost-estimation equations (Direct non-medical costs)**				
$\begin{array}{ll} Cost_{direct\;non-medical} & =\sum TC+FC+VDC\\ & =\sum \left(C_{ts}\times N_{s}+C_{td}\times N_{d}\right)+\left(D_{ip}\times C_{fc}\times N_{c}\right)+\left(C_{vd}\times N_{vd}\right) \end{array}$				

* after adjusting to 2017 value. ^a^ household survey on a sample of RTI casualties conducted by NIRC, 2018. ^b^ consensus of the researchers working in the NIRC. ^c^ assumption that each serious injury requires a caregiver.

**Table 3:** Base case values and equations for cost calculation (productivity costs).

Cost Items	Measure	Unit	Value	Sources
Short-term absenteeism from work (SA)	*D_sas_* = Duration of absence from work due to serious injury (days recovering in the hospital or at home)	Days	20	[34]
	*W_sas_* = Daily wage rate of persons seriously injured *	USD	3.43	[42]
	*N_sas_* = Number of people seriously injured	Number	4250	[33]
	*D_sac_* = Number of days spent by a caregiver for serious injury	Number	8	[34]
	*W_sac_* = Daily wage rate of a caregiver of persons seriously injured *	USD	3.43	[42]
	*D_sam_* = Duration of absence from work due to minor injury (days recovering in the hospital or at home)	Days	3	[34]
	*W_sam_* = Daily wage rate of people with a minor injury *	USD	3.43	[42]
	*N_sam_* = Number of people with minor injuries	Number	8290	[33]
Long-term disability (LD)	*n* = Average number of years lost	Years	40 ^a^	[33,47]
	*N_ld_* = Number of long-term disabled	Number	2646 ^b^	[33,35]
	*I_ld_* = Annual income of long-term disabled *	USD	658.41	[42]
	*I*_cld_ = Annual income of caregiver *	USD	658.41	[42]
	*N_cld_* = Number of caregiver	Number	706 ^c^	[35]
Premature death (PD)	*n* = Average number of years lost	Years	40 ^a^	[33,47]
	*I_pd_* = Annual income of victims of road crashes *	USD	658.41	[42]
	*N_pd_* = Number of premature deaths	Number	2384	[33]
**Cost-estimation equations (Productivity loss)**				
$\begin{array}{ll} Cost_{productivity\;loss} & =\sum SA+LD+PD\\ & =\sum \left[\{(D_{sas}\times W_{sas}+d_{sac}\times W_{sac})\times N_{sas}\}+\{(D_{sam}\times W_{sam})\times N_{sam}\}\right]\\ & +(\sum_{0}^{n}\frac{I_{Id}}{{\left(1+r\right)}^{n}}\times N_{Id}+\sum_{0}^{n}\frac{I_{cId}}{{\left(1+r\right)}^{n}}\times N_{cId}+\sum_{0}^{n}\frac{I_{pd}}{{\left(1+r\right)}^{n}}\times N_{pd}) \end{array}$				
r = discount rate of 3.0% (r = 0.03)				

* after adjusted to 2017 value. ^a^ life expectancy (70 years)—the average age of those died/disabled in a road crash (30 years). ^b^ long-term disabilities occur in approximately 21.1% of non-fatal RTIs in Nepal. The number of non-fatal RTIs in 2017 was the sum of serious and minor RTIs (12,540) from the Nepal Police record. ^c^ 26.7% of long-term disabled (N_ld_) need lifelong help to perform normal activities (daily living and transport).

**Table 4:** Total economic burden of road traffic injuries (RTIs) in Nepal in 2017.

Type of Cost	Number of RTI Cases	Cost (USD, Thousands)	Per Cent *
Direct medical costs		1497.26	**1.22%**
Inpatient service	4250	298.04	19.91%
Outpatient service	12,540	16.13	1.08%
Diagnostic services		233.27	15.58%
X-rays	4250	22.27	
Blood and urine investigations	4250	42.08	
CT scan	4250	165.21	
ECG	4250	3.71	
Doctors’ fee	12,540	269.72	18.01%
Medication		680.10	45.42%
Serious injuries	4250	610.10	
Minor injuries	8290	70.0	
Direct non-medical costs		11,499.82	**9.36%**
Transportation	6634	57.15	0.50%
Food	4250	172.83	1.50%
Vehicle damage	7708	11,269.84	98.0%
Productivity loss costs		91,566.69	**74.52%**
Premature death	2384	37,851.93	41.34%
Long-term disability (including the cost of caregiver)	2646	53,221.34	58.12%
Short-term absenteeism from work		493.42	0.54%
Serious injuries (including the cost of caregiver)	4250	408.13	
Minor injuries	8290	85.30	
Pain, grief and suffering costs		18,313.34	**14.90%**
**Total costs**		**122,877.11**	**100.0%**

* % in bold refer to the cost percentages according to type (e.g., direct medical costs).

**Table 5:** Sensitivity analysis.

Uncertain Variables	Cost (USD, Millions)
Direct medical costs (Base case)	1.50
Cost per inpatient bed-day in tertiary (super-speciality) hospitals	1.59
Doctors’ fee (private hospitals)	1.73
Doctors’ fee (Government hospitals)	1.34
Direct non-medical costs (Base case)	11.50
Administration costs	11.78
Productivity costs (Base case)	91.57
10.0% discount rate (premature death)	70.63
Average amount of working years lost due to premature death considering the age at retirement in Nepal	84.74
10.0% discount rate (long-term disabled)	62.13
